# Ecological and Anthropogenic Drivers of Hairtail Catch Distribution: A Spatial Analysis of the Southern Coastal Waters of South Korea

**DOI:** 10.3390/ani15172472

**Published:** 2025-08-22

**Authors:** Jongoh Nam, Cheolhyung Park, Jingon Son, Ohmin Kwon, Mingyeong Jeong, Moonsuk Lee

**Affiliations:** 1 Division of Marine & Fisheries Business and Economics, Pukyong National University, Busan 48513, Republic of Korea; namjo1234@pknu.ac.kr (J.N.); chpark@pknu.ac.kr (C.P.); 2 Resource & Environmental Economics Research Institute, Pukyong National University, Busan 48513, Republic of Korea; alrm7566@naver.com; 3 Ocean Policy Research Center, Korea Institute of Ocean Science and Technology (KIOST), Busan 49111, Republic of Korea; leems@kiost.ac.kr

**Keywords:** hairtail, grid data, spatial autocorrelation, spatial panel model

## Abstract

This study examined the spatial distribution and environmental determinants of hairtail (*Trichiurus lepturus*) catch volumes in the southern coastal waters of South Korea, employing a Spatial Durbin Model (SDM) based on grid-level data collected from 2020 to 2022. Key explanatory variables included chlorophyll-a concentration, dissolved oxygen, salinity, sea surface temperature, and fishing effort. Spatial autocorrelation was confirmed through Moran’s I test, justifying the application of a spatial econometric framework. Among the environmental factors, salinity exhibited the strongest positive direct effect on catch volumes, whereas dissolved oxygen consistently showed a negative effect. Chlorophyll-a concentration exhibited significant positive effects both within local grids and in neighboring areas. Sea surface temperature also had a modest but significant direct effect on catch volumes. Additionally, higher fishing effort was associated with increased catch volumes, emphasizing the spatial impact of human activities on fishery resources. These findings reveal that hairtail tend to aggregate in high-salinity, low-oxygen environments and respond to seasonal oceanographic variations. Overall, the results highlight the value of spatial econometric models in fisheries research by revealing how environmental and anthropogenic factors influence fish catch through both direct and indirect effects. The spatial framework offers deeper insight into the mechanisms driving hairtail distribution, particularly in ecologically complex regions like the Jeju Strait.

## Simple Summary

Hairtail (*Trichiurus lepturus*) is a commercially important fish species in South Korea, serving as both a vital food source and a major contributor to the local fishing industry. However, catch rates vary spatially and temporally in response to oceanographic conditions, including water temperature, dissolved oxygen levels, salinity, and food availability. In this study, we investigated the spatial and seasonal distribution of hairtail catches across the southern coastal waters of South Korea. We applied a spatial analytical approach that accounts for both site-specific conditions and the interactions among neighboring areas. Our results indicated that hairtail abundance was positively associated with areas of higher salinity and lower oxygen concentrations. Furthermore, regions with elevated phytoplankton biomass, an essential food source for smaller marine organisms, were found to enhance hairtail presence in adjacent waters. These findings advance our understanding of the species’ habitat preferences and environmental responses, providing insights that can inform more effective and sustainable fisheries management strategies. By integrating spatial mapping and environmental data, this research offers critical information for shaping future fishing policies and conserving key fishery resources such as hairtail.

## 1. Introduction

Marine ecosystems are shaped by intricate interactions between a wide array of environmental and anthropogenic factors, all of which exert significant influence on the distribution and dynamics of fishery resources [1]. Hairtail (*Trichiurus lepturus*) is one of the most commercially important species in South Korea, serving a pivotal role in both the ecological balance of marine environments and the economic value of fisheries. Therefore, the management and conservation of hairtail stocks have garnered significant attention from the Korean government [2].

Hairtail are primarily distributed in the southwestern waters off Jeju Island during the first quarter of the year. As sea temperatures rise in the second quarter, they begin migrating northward along the western coast of Korea. In the third quarter, spawning is reported to occur in the southern and southwestern waters of Korea. By the fourth quarter, declining temperatures are believed to prompt their return to the southwestern waters off Jeju Island for overwintering [2,3]. Accordingly, hairtail are primarily distributed in the southern and western coastal waters of Korea, as well as the East China Sea, and exhibit year-round migratory behavior [4]. Therefore, this study aims to quantitatively analyze the relationships between hairtail catch volumes and various oceanographic factors such as temperature, salinity, and food availability to understand the spatial distribution characteristics of hairtail resources. Against this background, a systematic analysis based on spatiotemporal data encompassing ecological, environmental, and fisheries information is essential to comprehensively assess the spatial distribution and population dynamics of hairtail [5,6].

In recent years, the combined effects of climate change, shifts in oceanographic conditions, and increasing human activity have introduced considerable uncertainty into hairtail habitat dynamics and resource availability [7]. These changes pose new challenges for fisheries governance, as conventional analytical approaches often fail to effectively capture the complexity of spatial interactions inherent in marine ecosystems. In this context, spatial data-based methodologies—particularly spatial panel analysis—have emerged as powerful tools for understanding the spatiotemporal variability of marine resources [1,8]. Particularly, spatial panel analysis enables the estimation of spillover effects, wherein explanatory or dependent variables in a given marine grid influence the dependent variables in neighboring grids. This feature makes it especially well-suited for analyzing the distribution of migratory species such as hairtail, as it allows for a more precise assessment of the various factors influencing their spatial dynamics.

The objective of this study is to empirically examine the spatial interdependence of hairtail catch volumes using a spatial panel model while identifying the key environmental and anthropogenic factors that drive these variations. By adopting this approach, the study aims to elucidate how spatial patterns in hairtail abundance are influenced by marine environmental conditions and fishing activities, thereby highlighting the necessity of incorporating spatial structures into fisheries management policy design.

Spatial econometrics has been widely applied across various fields. In environmental economics, Espoir and Sunge [9] employed the Spatial Durbin Model (SDM) to examine how economic growth and energy consumption in African countries influence CO_2_ emissions within both domestic and neighboring nations. Jung et al. [10] applied a Bayesian spatial regression model to examine spatial correlations between urban structure and air pollution in South Korea.

In urban and regional economics, Li et al. [11] employed spatial panel analysis to examine the spatial spillover effects of transportation infrastructure on urban resilience across major metropolitan areas in China. Park and Yun [12] applied a spatial panel model to identify the social determinants of residential electricity consumption in local municipalities of South Korea.

In real estate economics, spatial econometric techniques have been incorporated into traditional hedonic pricing frameworks to enhance the estimation of spatial dependencies observed in housing markets. Osland [13] applied various spatial models to the Norwegian housing market and analyzed how spatial autocorrelation and heterogeneity influence model performance.

Thus, spatial econometrics offers significant policy implications by enabling not only comparisons across regions but also the quantitative analysis of spatial interactions, spatial dependence, and spillover effects among neighboring areas. As a result, it has been widely applied across various fields of economics. However, studies that apply spatial econometric models to quantitatively examine the relationships between the spatial distribution of marine species and environmental variables remain extremely limited. For instance, Kim et al. [14] analyzed the relationship between coastal fisheries production in South Korea and climate-related factors such as sea surface temperature and salinity using a spatial panel model. However, the data employed in their study were not based on observed marine grid-level measurements, but rather on regionally aggregated and reconstructed data. This presents a key distinction from the present study, which utilizes spatially explicit grid-level observations in marine environments.

Although several marine studies have explored species–environment relationships using multivariate methods like redundancy analysis (RDA) [15,16,17], those analyses often offer limited spatial inference. A notable example is Shi et al. [5], who applied a random forest species distribution model (SDM) to investigate *Trichiurus japonicus* in the East China Sea and south-central Yellow Sea, identifying habitat suitability based on bottom temperature, depth, and surface salinity. However, their approach primarily characterized the environmental thresholds associated with fishing grounds rather than quantitatively estimating spatial spillover effects. This key methodological distinction marks a clear divergence from the spatial econometric framework applied in the present study.

This study advances the marine and fisheries literature by leveraging grid-level catch data of commercially important species to delineate the spatial distribution of fishery resources and employing spatial econometric techniques to investigate the linkages between catch volumes, marine environmental conditions, and fishing effort at a high spatial resolution. The insights gained extend beyond resource-specific stock assessments to inform broader fisheries management frameworks and provide a robust empirical basis for policy-making geared toward sustainable marine resource governance.

The structure of this paper is as follows: Section 2 outlines the research methodology for the spatial panel model. Section 3 presents the empirical results derived from the spatial econometric analysis. Finally, Section 4 and Section 5 discuss the key findings and policy implications and offer concluding remarks. Through this structure, this study seeks to contribute to the development of spatially adaptive fisheries management policies by providing empirical evidence based on a spatially explicit analysis of the distribution of hairtail resources.

## 2. Materials and Methods

### 2.1. Data Source and Management

In this study, the dependent variable is the catch volume of hairtail, measured using monthly grid-level data provided by the National Federation of Fisheries Cooperatives for South Korean coastal waters between 2020 and 2022. As Jeju Island and the southern coast are the primary production areas for hairtail, the analysis focuses on the marine zones of Busan, Gyeongsangnam-do, Jeollanam-do, and Jeju, following the regional classifications defined in local marine spatial plans. The spatial resolution of the dataset is set at 1 min and 30 s per grid.

The model includes five explanatory variables: four environmental indicators and one proxy variable representing fishing effort, each of which is hypothesized to influence hairtail catch volumes. The ecological indicators, chlorophyll-a (Chl-a), dissolved oxygen (DO), salinity (Sal), and sea surface temperature (SST), were selected due to their critical roles in determining fish habitat suitability and spatial distribution [15,16,17,18]. Chl-a is a representative indicator of phytoplankton biomass and reflects the productivity of lower trophic levels, which is expected to influence the habitat suitability and aggregation of hairtail. Sal and SST are variables closely associated with seasonal changes and ocean currents, and are considered important factors in explaining the seasonal migration and aggregation patterns of hairtail. Lastly, DO was included to analyze the response of fish aggregation and distribution to variations in oxygen availability.

Chl-a and SST data were derived from satellite-based observation, specifically the Monthly Composite products of the Aqua MODIS sensor provided by the National Oceanic and Atmospheric Administration (NOAA) [19]. In contrast, Sal and DO data were obtained from in situ measurements conducted through Korea’s Line Oceanographic Observation and Marine Environmental Monitoring programs [20,21]. Fishing effort was quantified as the number of vessels operating within each marine grid, based on records from the National Federation of Fisheries Cooperatives. A higher number of vessels within a given grid was interpreted as indicative of greater fishing activity in that area.

All data spanning the three years were converted into quarterly observations. The catch volume of hairtail and the number of fishing vessels were aggregated into quarterly totals, while Chl-a and SST were converted to quarterly average values. For example, the first-quarter catch volume of hairtail represents the total catch volume for January, February, and March. In contrast, the first-quarter Chl-a value is calculated as the average of monthly Chl-a concentrations during the same period. However, due to the nature of the observational data used for Sal and DO, measurements were available only four times per year—specifically in February, May, August, and November. Accordingly, each of these measurements was used as a representative value for the corresponding quarter.

Descriptive statistics for the dataset are summarized in Table 1. The average grid-level catch volume of hairtail peaked in the fourth quarter, reaching approximately 3030 kg, while the lowest average was recorded in the second quarter at 574 kg. The standard deviation of catch volumes was also highest in the fourth quarter (91,802 kg), indicating substantial spatial variability, and lowest in the second quarter (4124 kg). Chl-a concentrations exhibited relatively minor variation across quarters, with mean values ranging from 1.32 to 1.38 mg/m^3^. However, the standard deviation was highest in the third quarter, at 2.19, presenting greater spatial variability during this period. DO concentrations were highest in the first quarter, averaging 6.52 mg/L, and lowest in the third quarter, at 5.50 mg/L. Sal was highest in the first quarter, with an average of 33.86 PSU, and decreased to its lowest value of 31.10 PSU in the third quarter. SST reached its maximum in the third quarter, at 25.01 °C, reflecting peak summer conditions, and its minimum in the first quarter, at 13.23 °C, consistent with winter temperatures. The number of fishing vessels per grid followed a seasonal pattern as well, with the highest average observed in the fourth quarter, at 596 vessels, and the lowest in the first quarter, at 411 vessels.

To stabilize the variance in the time-series data, all continuous variables were transformed into their natural logarithmic forms. The estimated coefficients from the log–log model specification can be interpreted as elasticities, indicating the percentage change in the dependent variable associated with a 1% change in the corresponding independent variable [22,23].

**Figure 1:**
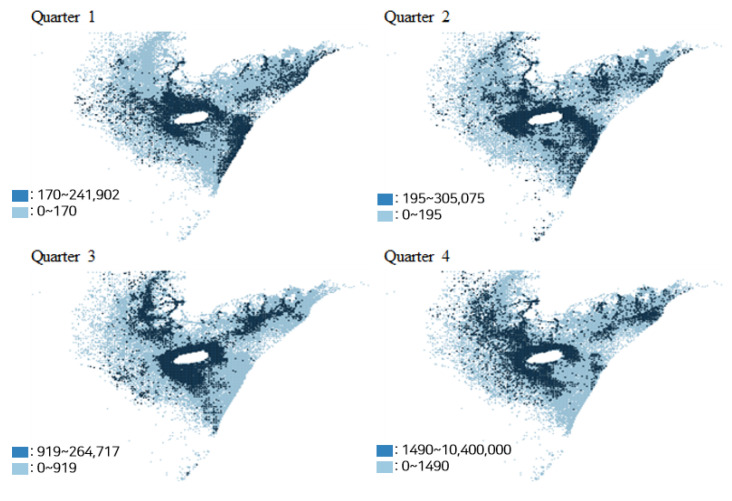
Catch distribution of hairtail by quarter. Note: Two categories are presented in the legend, separated at the 75th percentile.

Figure 1 illustrates the quarterly average distribution of hairtail catch volumes over three years (2020–2022) in the southern coastal waters of South Korea. The central white circular area in the figure denotes the location of Jeju Island. Catch volumes are depicted using a blue color gradient, with lighter shades representing lower catch levels and darker shades indicating higher catch levels.

The spatial distribution of hairtail catch reveals a distinct seasonal pattern. In the first quarter, catches are predominantly concentrated in the waters surrounding Jeju Island and along the Japanese Exclusive Economic Zone. During the second and third quarters, fishing activity shifts toward the coastal areas of the Yellow Sea and the southern mainland coast of South Korea. In the fourth quarter, high catch volumes reappear in the western offshore waters of Jeju Island.

This seasonal pattern corresponds to the known migratory behavior of hairtail, which typically inhabit the southwestern waters of Jeju Island from January to March, migrate northward toward the Yellow Sea coast starting in April, and return to the southwestern waters near Jeju Island from September onward [4].

Prior to conducting the spatial panel model analysis, it was necessary to assess the presence of multicollinearity among the independent variables. To this end, variance inflation factors (VIFs) were calculated for each explanatory variable as a diagnostic measure. Multicollinearity can distort coefficient estimates, leading to inaccurate inference or the appearance of insignificance in variables that are, in fact, significant.

Specifically, high correlations among explanatory variables can inflate the standard errors of the estimated coefficients, thereby reducing their statistical significance. A VIF quantifies the degree to which multicollinearity increases the variance of the estimated coefficients. Multicollinearity is generally considered problematic when the VIF exceeds 10 or when its reciprocal, 1/VIF, is less than 0.1 [24].

The diagnostic results showed that all VIF values were considerably below the threshold of 10, indicating a low degree of multicollinearity among the explanatory variables (Table 2).

### 2.2. Spatial Autocorrelation

Spatial autocorrelation describes the degree to which neighboring spatial units exhibit statistical similarity [25]. The concept is rooted in Tobler’s First Law of Geography, which states that “everything is related to everything else, but near things are more related than distant things” [26].

Among the various methods for detecting spatial autocorrelation, Moran’s I statistic is the most widely used [27]. Moran’s I quantifies the degree of spatial similarity by calculating a correlation coefficient based on the attribute values of neighboring observations.

(1) $$I=n\sum_{i}\sum_{j}w_{ij}(x_{i}-\bar{x})(x_{j}-\bar{x})/W\sum_{i}{\left(x_{i}-\bar{x}\right)}^{2}$$

In the formula, n denotes the total number of spatial units (grids), and $x_{i}$ and $x_{j}$ represent the attribute values of variable x in grid i and grid j, respectively. The term $\bar{x}$ indicates the average value of x across all grids. The spatial weight $w_{ij}$ captures the structure of spatial proximity or connectivity between grids i and j, and W  denotes the sum of all spatial weights in the matrix. The value of Moran’s I ranges between −1 and +1. A value close to +1 indicates strong positive spatial autocorrelation, meaning that neighboring spatial units tend to have similar attribute values. Conversely, a value close to −1 suggests negative spatial autocorrelation, where neighboring units exhibit dissimilar characteristics. A Moran’s I value near 0 implies spatial randomness, indicating that the variable of interest does not show any statistically significant spatial pattern.

In the context of this study, a Moran’s I value approaching +1 implies that hairtail catch volumes are similar across neighboring marine grids [28]. Conversely, a value closer to −1 indicates that areas with high catch volumes are located adjacent to areas with low catch volumes [28]. A Moran’s I value near 0 suggests a random spatial distribution of catch volumes, indicating the absence of significant spatial dependence.

### 2.3. Spatial Panel Model

Spatial regression models are designed to account for spatial autocorrelation that may arise when cross-sectional or panel data consist of observations with spatial attributes. According to Anselin [29], spatial dependence may be present when neighboring regions share similar unobserved shocks or when neighboring values influence the dependent variable. Ignoring such spatial effects during the estimation process can lead to biased and inconsistent parameter estimates because spatial dependence violates the classical assumptions underlying the ordinary least squares (OLS) estimator.

To address these issues, various spatial econometric models—such as the spatial autoregressive (SAR) model, the spatial error model (SEM), and the spatial Durbin model (SDM)—have been developed. Among these, the present study employs the SDM to capture potential spatial spillover effects of the explanatory variables on neighboring regions. The SDM extends the SAR model by incorporating spatial lags of both the dependent and independent variables, allowing for a more comprehensive representation of spatial interactions.

(2) $$y_{t}=\rho Wy_{t}+X_{t}\beta +WX_{t}\theta +\iota_{n}\alpha +\mu +\varepsilon_{t},\varepsilon_{t}\sim N(0,\sigma^{2}I_{n})$$

Each variable in this model is structured as panel data consisting of n cross-sectional units over time. Specifically, $y_{t}$ is an n×1 vector representing hairtail catch volumes for each region at time t, and $X_{t}$ is an n×k matrix of explanatory variables. The vector $\iota_{n}\alpha$ is an n×1 vector of ones corresponding to the intercept, while μ denotes unobserved region-specific effects and $\varepsilon_{t}$ is the error term.

The spatial relationship among regions is captured by the spatial weight matrix W, an n×n matrix where each element $w_{ij}$ reflects the spatial dependence between regions i and j. The matrix is assumed to be time-invariant and row-standardized such that the sum of weights in each row equals one. The coefficient ρ captures the degree of spatial dependence in the dependent variable.

If θ=0, the model reduces to the SAR model, which accounts only for spatial dependence in the dependent variable. Conversely, if θ+ρβ=0, the model simplifies to the SEM, which captures spatial dependence exclusively in the error term. The SDM serves as a more general specification, allowing for spatial dependence in both the dependent variable and the explanatory variables [30,31].

The SAR and SEM can be expressed as:

(3) $$SAR:y_{t}=\rho Wy_{t}+X_{t}\beta +\iota_{n}\alpha +\mu +\varepsilon_{t},\varepsilon_{t}\sim N(0,\sigma^{2}I_{n})$$

(4) $$SEM:y_{t}=X_{t}\beta +\iota_{n}\alpha +\mu +u_{t},u_{t}=\lambda Wu_{t}+\varepsilon_{t},\varepsilon_{t}\sim N(0,\sigma^{2}I_{n})$$

Similarly to conventional panel models, spatial panel models can be estimated using either fixed-effect (FE) or random-effect (RE) specifications. The FE model treats μ as a set of unit-specific dummy variables, while the RE model assumes μ to be a random variable uncorrelated with the error term $\;\varepsilon_{t}\;$ [32]. To determine the appropriate specification, a Hausman test incorporating spatial effects was conducted. The test results supported the FE model, which was subsequently employed for estimation (Table 3).

In the presence of spatial dependence in either the dependent or independent variables, a marginal change in an independent variable for a given unit may influence not only the unit itself but also neighboring units. In such cases, the estimated coefficients no longer directly reflect total marginal effects. LeSage [33] proposed decomposing the total effect into direct and indirect effects. The direct effect measures the effect of a change in an independent variable on the dependent variable within the same spatial unit. In contrast, the indirect effect, referred to as the spatial spillover effects, measures the effect of that change on the dependent variables of neighboring units through spatial interdependencies.

To derive these effects, Equation (2) can be reformulated as Equation (5):

(5) $$\begin{array}{cc} y_{t}={\left(I_{n}-\rho W\right)}^{-1} & \iota_{n}\alpha +{\left(I_{n}-\rho W\right)}^{-1}\left(X_{t}\beta +WX_{t}\theta \right)+{\left(I_{n}-\rho W\right)}^{-1}\varepsilon_{t}^{*}\\ & ={\left[\sum_{k=1}^{k}S_{k}(W)x_{k}\right]}_{t}+{\left(I_{n}-\rho W\right)}^{-1}\iota_{n}\alpha +{\left(I_{n}-\rho W\right)}^{-1}\varepsilon_{t}^{*} \end{array}$$

Here, it is assumed that $S_{k}\left(W\right)={\left(I_{n}-\rho W\right)}^{-1}\left(I_{n}\beta_{k}+W\theta_{k}\right)$, and $\varepsilon_{t}^{*}$ denotes the composite error term, which includes both the idiosyncratic error $\varepsilon_{t}$ and the fixed effects μ. To compute the marginal effect of the k^th^ explanatory variable $x_{ik}$ for region i at time t, both sides of the model are partially differentiated with respect to $x_{k}$, yielding the following expression, as shown in Equation (5).

(6) $${\left[\frac{\partial y}{{\partial x}_{k}}\right]}_{t}=S_{k}\left(W\right)={\left(I_{n}-\rho W\right)}^{-1}\left[\begin{array}{ccc} \beta_{k} & \ldots & w_{1n}\theta_{k}\\ \vdots & \ddots & \vdots \\ w_{n1}\theta_{k} & \ldots & \beta_{k} \end{array}\right]$$

In this framework, the direct effect is defined as the average of the diagonal elements of the matrix $s_{k}\left(w\right).$ The total effect is calculated as the average of the row (or column) sums of $s_{k}(w)$ across spatial units. The indirect effect is then derived as the difference between the total and direct effects.

LeSage and Pace [30] proposed a simulation-based approach to derive the distributions of the direct and indirect effects using the variance–covariance matrix obtained from the maximum likelihood estimates. Following this approach, the present study calculates the point estimates of the direct and indirect effects and performs statistical significance tests based on their simulated distributions.

## 3. Results

### 3.1. Spatial Autocorrelation in Hairtail Catch Volumes

To examine the spatial autocorrelation structure of the dependent variable, namely, the hairtail catch volume, Moran’s I tests were conducted using quarterly data over three years (Table 4). The spatial weight matrix was constructed based on an inverse distance approach, using centroid-to-centroid distances between grids. In this matrix, the strength of spatial interaction increases as the distance between grid pairs decreases, and diminishes rapidly as the distance grows, thereby providing a more accurate representation of distance-based spatial interactions within the marine environment. In addition to the inverse distance matrix, two alternative spatial weight matrices were considered: an inverse distance matrix with a 50 km cutoff and a Queen contiguity matrix. To determine the most appropriate specification, spatial autocorrelation analysis was first conducted, revealing that the inverse distance matrix consistently produced the highest Chi-squared statistics. Estimation results from the Spatial Durbin Model (SDM) further demonstrated that coefficient estimates were most statistically significant when using the inverse distance matrix. Model comparison using AIC (178,278.60) and BIC (178,385.00) also indicated that this matrix provided the best overall fit. From an ecological standpoint, considering the migratory behavior of hairtail, the inverse distance matrix, capable of capturing spatial influence across the entire study area, was deemed most appropriate for representing the species’ movement and habitat utilization patterns.

The results of the Moran’s I test revealed statistically significant positive spatial autocorrelation at the 1% significance level across all quarters. All Moran’s I values were greater than zero, suggesting that hairtail catch volumes are spatially clustered. Specifically, areas with high (or low) catch volumes tend to be surrounded by neighboring grids exhibiting similarly high or low values. These findings suggest a spatially dependent pattern in hairtail distribution, characterized by distinct clustering of catch intensity across neighboring marine grids.

However, it is noteworthy that Moran’s I value in the fourth quarter (0.003) is substantially lower than that of the third quarter (0.444). This pattern can be understood in the context of hairtail annual migratory behavior, as illustrated in Figure 1. During the third quarter, hairtail densely aggregate for spawning in the southern and southwestern coastal waters of Korea, resulting in highly clustered catch distribution in specific areas. In contrast, during the fourth quarter, hairtail begin their return migration toward the southwestern offshore waters near Jeju Island for overwintering. As a result, the spatial distribution of catch volumes becomes more dispersed and extends farther offshore, leading to a much weaker spatial clustering effect.

### 3.2. Global vs. Local Spatial Effects

Global spatial effects refer to situations in which the value of the dependent variable in one region ($Y_{i}$) influences the outcomes across the entire study area ($Y_{j}$). In the context of this study, for instance, a change in hairtail catch volume in one marine grid may affect catch volumes throughout all neighboring regions. In contrast, local spatial effects are characterized by the influence of $Y_{i}$ being confined to a limited set of neighboring regions, with the spatial dependence diminishing over distance.

LeSage [33] classified spatial spillover effects into two categories: local and global. Local spillover effects, which influence only neighboring units, are appropriately modeled using the Spatial Durbin Error Model (SDEM). In contrast, global spillover effects, including feedback effects that permeate the entire spatial system, are captured by the Spatial Durbin Model (SDM).

To determine the most appropriate model for the data, both the SDM and SDEM were estimated, reflecting global and local spillover processes, respectively. Model performance was evaluated using standard information criteria, specifically the Akaike Information Criterion (AIC) and the Bayesian Information Criterion (BIC), to determine the best-fitting model (Table 5).

The results indicated that the SDEM yielded lower values for both the AIC and BIC, suggesting that the model accounting for local effects provides a better statistical fit. However, the absolute differences in information criteria between the two models were relatively modest—approximately 1200 in both AIC and BIC—suggesting that, although the statistical distinction is notable, it may not be decisive on its own.

As noted by Burnham and Anderson [34], model selection should not be based solely on differences in information criteria, but should also consider the interpretability of the model and its consistency with theoretical expectations.

Given that the primary aim of this study is to identify and interpret spatially structured direct and indirect effects of marine environmental variables on hairtail catch volumes, the SDM is theoretically more appropriate due to its capacity for structural decomposition of spillover effects. As noted in the introduction, hairtail is a typical migratory species that moves extensively across the southern and western waters and areas surrounding Jeju Island in response to changes in sea temperature, spawning seasons, and other conditions. This wide-ranging mobility suggests that environmental conditions or fishing activity in one region may influence resource density or catch levels even in distant areas. Although the SDEM exhibited slightly lower BIC values, the difference is minimal at approximately 0.68% and does not justify a purely mechanical model selection based solely on information criteria. Accordingly, the SDM, which accounts for global interactions and feedback effects, is considered the more appropriate model, in line with the theoretical framework proposed by LeSage and Pace [30].

Therefore, despite the SDM having slightly higher information criterion values, it was selected for its theoretical consistency and superior interpretability.

### 3.3. SDM Estimation and Model Validation

The estimation results for hairtail catch volumes are presented in Table 6. Both the spatial autoregressive coefficient (ρ) and the coefficient for the spatially lagged independent variables (θ) were statistically significant at the 1% level across all spatial panel model specifications. These findings confirm the existence of significant spatial dependence in hairtail catch volumes.

Table 7 summarizes the results of the Likelihood Ratio (LR) and Wald tests conducted to evaluate whether the SDM can be simplified to more basic spatial models, such as the SAR, SEM, Panel, or SLX models.

First, the LR test was performed under the null hypothesis $H_{0}:\rho =0$, which tests whether spatial dependence exists solely through the spatially lagged explanatory variables, specifically whether the SDM can be reduced to the SLX model. The null hypothesis was rejected at the 1% significance level, indicating that spatial autocorrelation in the dependent variable is statistically significant. This result suggests that the SLX model is an inadequate specification, as it fails to account for spatial dependence in the dependent variable.

Next, the hypothesis $H_{0}:\theta =0$, which tests whether spatial spillover effects from explanatory variables are absent, was also rejected at conventional significance levels. This finding indicates that the SDM cannot be simplified to the SAR model, as spatial lags of the explanatory variables contribute significantly to the model specification.

Furthermore, the joint null hypothesis $H_{0}:\rho =0,\theta =0$ was rejected, indicating that the Spatial Durbin Model (SDM) cannot be reduced to a non-spatial panel model. This result underscores the necessity of accounting for spatial dependence in both the dependent variable and the spatially lagged explanatory variables. Additionally, the Wald test for the structural reduction condition θ+ρβ=0, as proposed by LeSage and Pace [30], was significantly rejected, indicating that the SDM and SEM are not structurally equivalent.

Collectively, these results support the SDM as the most appropriate specification, as it captures both direct spatial dependence and spatial spillover effects. The series of diagnostic test results confirms that the dataset used in this study exhibits spatial and structural dependencies, thereby justifying the adoption of the SDM as the most appropriate model. This reinforces the importance of interpreting the results in terms of both direct and indirect spatial effects.

According to the SDM estimation results presented in Table 6, most marine environmental variables exert statistically significant effects on hairtail catch volumes. In particular, Sal, SST, and Chl-a exhibit significantly positive coefficients, indicating that increases in these variables are associated with higher catch volumes. Conversely, DO is associated with a statistically significant negative coefficient, suggesting that hairtail may be more densely distributed in water masses with relatively lower oxygen concentrations.

In addition, most of the spatially lagged terms of the environmental variables (W*X), except DO, were estimated with negative coefficients, implying the potential presence of spatial substitution or clustering effects, whereby an increase in the level of a variable in one grid may lead to a decrease in catch volumes in neighboring grids.

### 3.4. Marginal Effects: Direct, Indirect, and Total

It is important to note, however, that the coefficients estimated from the SDM do not directly represent marginal effects. Therefore, following the methodology proposed by LeSage and Pace [30], the results were decomposed into direct, indirect, and total effects. The marginal effects derived from the SDM estimation are summarized in Table 8. The standard errors of marginal effects were calculated using the Delta method. The Delta method is a theoretically valid and computationally efficient technique based on the Taylor series expansion, commonly used for estimating standard errors of nonlinear functions.

Table 8 presents the marginal effects estimated from the SDM, decomposing the total effects of each marine environmental variable on hairtail catch volumes into direct, indirect, and total effects.

Sal exhibited the highest direct effect among all variables, indicating that a 1% increase in salinity leads to a 14.47% increase in hairtail catch volume within the same grid. However, the indirect effect was negative (−6.44%), suggesting that higher salinity in one grid may reduce catch volumes in neighboring grids. The total effect of Sal, at 8.03%, was the largest among all variables, indicating that it was the most significant factor. According to Shi et al. [5], hairtail in the East China Sea exhibited the highest habitat suitability at surface salinity levels around 31.2 PSU and bottom salinity levels near 33.3 PSU. Similarly, Liu and Cheng [35] found that the major distribution areas of hairtail in the East China Sea were predominantly associated with salinity levels around 34 PSU, particularly during spring and summer when salinity conditions were most stable and concentrated. In addition, in the Jeju Strait, hairtail fishing grounds were reported to form intensively in areas where salinity ranges between 33 and 34 PSU [36]. These findings collectively suggest that hairtail tends to aggregate in marine areas with salinity within a specific optimal range. According to the theory of density-dependent habitat selection, individuals tend to aggregate in habitats with higher suitability, resulting in increased population density in the central area and relatively lower or declining densities in surrounding regions [37]. In the context of this study, if salinity in grid A increases and reaches the optimal level preferred by hairtail, individuals may move toward grid A, thereby increasing the catch volume in that area (direct effect). Conversely, such aggregation can lead to reduced catch volumes in neighboring grids (e.g., B and C), resulting in a negative indirect effect.

DO showed significant negative effects across both spatial dimensions, with a direct effect of −5.76% and an indirect effect of −11.76%, resulting in a total effect of −17.53%. This suggests that increases in oxygen concentration are associated with substantial reductions in catch volumes, both locally and in surrounding areas.

Chl-a exhibited a direct effect of 0.60% and an indirect effect of 3.93%, resulting in a total effect of 4.53%, with a larger contribution from the indirect effect. This suggests that increases in primary productivity can exert positive influences not only within a grid but also across neighboring waters. The stronger indirect effect of Chl-a can be explained by the time lag inherent in the trophic transfer process. According to Apriliani et al. [38], an increase in Chl-a concentrations promotes the productivity of zooplankton, thereby forming a food chain that supports the growth of fish populations. However, their study also found that even when Chl-a levels increase, it takes time for fish to respond to this change due to the lag in trophic transfer. This is because plankton is not a direct food source for hairtail. In the southern coastal waters of Korea, anchovy (Engraulis japonicus) has been identified as the main prey species of hairtail. Anchovy is known as a representative secondary consumer that links both phytoplankton and zooplankton (lower trophic levels) with higher predators such as hairtail [39]. Therefore, the increase in Chl-a within a given grid is likely to have had a greater impact on hairtail catch volumes in neighboring grids, by enhancing the abundance of zooplankton and intermediary prey species, than within the grid itself. This supports the interpretation that the indirect effect of Chl-a exceeds its direct effect. Accordingly, the result supports the relevant literature [38,39] on the food web structure in the southern coastal waters of Korea, illustrating how primary productivity is propagated through intermediate trophic levels, namely zooplankton and anchovy, before reaching higher-level predators such as hairtail.

For SST, the direct effect was statistically significant at 0.46%, whereas the indirect effect was not statistically significant, resulting in a total effect of 0.45%.

Regarding fishing effort, measured by the number of vessels, the total effect was 0.56%, with the majority attributable to the direct effect (0.53%).

## 4. Discussion

This study followed a structured procedure for conducting spatial econometric analysis. First, spatial autocorrelation in hairtail catch volumes was assessed using Moran’s I, thereby confirming the necessity of applying a spatial econometric approach. Second, the SDM model was selected as the most appropriate model by comparing the SDEM model reflecting the regional ripple effect with the SDM model reflecting the global ripple effect. Third, various models, including SDM, SAR, SEM, SLX, and a non-spatial panel model, were estimated, and likelihood ratio and Wald tests were conducted to statistically validate the suitability of the SDM. Finally, a marginal effects analysis was performed to decompose the total effect of marine environmental and fishing effort variables on hairtail catch volumes into direct and indirect effects, thereby clearly illustrating spatial spillover mechanisms.

Several studies have examined the relationship between hairtail distribution and environmental factors, particularly in the East China Sea and the Jeju Strait [5,35,36]. However, these studies primarily provided descriptive interpretations of the conditions under which fishing grounds are formed. In contrast, this study is the first to employ the Spatial Durbin Model (SDM) to estimate not only spatial aggregation but also the direct and indirect effects of environmental variables on hairtail catch volumes in the East China Sea and the Jeju Strait. This approach allows for the analysis of aggregate dynamics and inter-grid ecological influences that cannot be identified through technical analysis, such as a random forest model or a mixed distribution-decomposition method. Furthermore, if spatial data at the ocean grid level becomes available for the East China Sea in the future, it would be valuable to compare the effects of salinity on hairtail distribution across regions and explore potential regional differences.

The marginal effects analysis provides robust empirical evidence that hairtail catch volumes are shaped by spatially structured ecological mechanisms. Among the examined variables, Sal emerged as the most influential factor, exerting the strongest positive direct effect while simultaneously exhibiting significant negative spillover effects. These findings are consistent with the ecological characteristics of hairtail described in the study by Kim and Rho [36], who noted that hairtail tend to aggregate in high-Sal water masses (33.7–34.5 PSU), often near thermohaline fronts and in deeper layers (~150 m). The presence of a strong positive direct effect alongside a negative indirect effect supports the hypothesis of a spatial substitution mechanism, wherein hairtail are concentrated within favorable water masses, resulting in lower resource density in neighboring grids.

DO was found to have a consistently negative effect on hairtail catch volumes in both local and neighboring grids. This suggests that hairtail may exhibit ecological traits enabling them to form aggregations even in environments with relatively low oxygen concentrations. Although DO has not been a central focus of previous ecological studies, the species’ tendency to aggregate along hypoxic boundary layers implies a certain degree of tolerance to low-oxygen conditions, a trait indirectly supported by the findings of this spatial econometric analysis.

Chl-a, a proxy for phytoplankton biomass and a key indicator of lower trophic-level productivity, exhibited statistically significant positive effects in both direct and indirect dimensions [38]. Locally, higher concentrations of Chl-a were associated with increased hairtail catch volumes, suggesting that elevated primary productivity promotes aggregation within the same area. Additionally, significant spillover effects indicated that productive areas might also enhance catch volumes in neighboring regions through a diffusion-based distribution mechanism. Although previous studies did not directly assess Chl-a in the Jeju Strait, reported fishing grounds near high-temperature and high-salinity fronts or localized upwelling zones suggest a potential ecological linkage with the spatial patterns identified in this study.

SST showed a statistically significant direct effect on hairtail catch volumes, although its overall effect was weaker than that of Sal or Chl-a. No significant spillover effects were observed, suggesting that SST may have a more localized effect on catch outcomes. Ecological studies, including that of Kim and Rho [36], have reported that in the Jeju Strait—particularly during summer and autumn—fishing grounds frequently formed along thermal fronts where SST ranged between 23.0 °C and 27.8 °C. These thermal structures form where warm offshore currents meet cooler coastal waters. This boundary, shaped by tidal mixing and freshwater inflows, often corresponds to areas of high biological productivity and prey abundance. While SST alone may not be a dominant driver of spatial variation in hairtail catches across the southern coast, it likely interacts with other oceanographic variables to shape localized ecological conditions. In this context, SST may serve as a meaningful variable for interpreting the regional characteristics of hairtail resources in dynamic oceanographic areas such as the Jeju Strait.

In addition to environmental variables, fishing effort, measured by the number of vessels, exhibited a statistically significant positive direct effect on hairtail catch volumes. Higher fishing effort within a grid was linked to increased catch volumes, underscoring the spatial alignment of fishing effort with resource distribution. Although no significant spillover effects were detected, the result highlights the importance of accounting for anthropogenic factors in spatial analyses of fishery resources, particularly in intensively exploited coastal waters.

Together, the findings imply that the dynamics of fish catches in marine ecosystems are shaped by both local environmental conditions and spatial interactions among neighboring areas. For species such as hairtail, which are highly sensitive to seasonal and hydrographic variability, understanding the spatial structure and interplay of environmental variables is essential for accurately capturing resource distribution patterns. By applying the SDM, this study systematically distinguished between direct and indirect effects, enabling a more nuanced analysis of spatial diffusion mechanisms among environmental drivers. This spatial approach improves our ability to explain hairtail distribution, particularly in ecologically complex regions such as the Jeju Strait, where oceanographic fronts and water mass boundaries frequently intersect [40].

Taken together, these findings provide not only ecological insights but also practical relevance for fisheries governance. These insights have direct implications for fisheries policy and management. Spatially informed strategies that incorporate both localized and spillover effects can enhance the accuracy of habitat forecasting and support adaptive zoning or conservation efforts in regions such as the Jeju Strait. Collectively, these findings provide a scientific basis for integrating spatial patterns into fisheries resource evaluation and habitat planning. In particular, the Korean government has been implementing the First National Marine Spatial Plan (2019–2028) to establish a strategic, nationwide framework for the integrated management and sustainable use of marine space. The analytical results of this study can serve as a scientific basis for assessing spatial characteristics of marine areas and for developing marine spatial plans, as they provide a quantitative understanding of the spatial distribution of fishery resources and their responses to changes in environmental conditions. Furthermore, these findings may contribute meaningfully to the development of region-specific, adaptive resource management strategies.

Nonetheless, the study has several limitations. First, although major oceanographic variables such as Chl-a, SST, Sal, and DO were included, key biological factors like the spatial distribution of prey species (e.g., anchovy and small yellow croaker) were not incorporated, limiting the ecological interpretation of catch variability. Future studies should incorporate biological variables, such as the abundance of mid-trophic organisms, to enable a more comprehensive and ecologically valid explanation of variations in hairtail catch volumes.

Second, the spatial scope of the analysis was confined to the broader southern coastal region, potentially underrepresenting localized ecological responses, particularly in distinct environments such as the Jeju Strait. Future research should consider integrating prey-related biological indicators and conducting more localized analyses that treat the Jeju coastal zone as a separate spatial regime.

Third, due to limited data availability in South Korea, the number of vessels was used as a proxy for fishing effort. No grid-level data are currently available that distinguish effort by operation time, gear type, or fishing method. However, actual fishing effort is influenced by these factors. The exclusion of these aspects may lead to an underestimation or overestimation of true fishing effort. Future research should aim to acquire disaggregated effort data that accounts for gear-specific fishing power and temporal variability. If such data become available, applying CPUE standardization techniques (e.g., Gavaris 1980) or gear-weighted adjustment factors would improve both the ecological accuracy and policy relevance of spatial analyses in fisheries science. 

Finally, this study did not incorporate several ecological and oceanographic fac-tors—such as seafloor topography, thermocline depth, and ocean currents—that may influence the spatial distribution of hairtail. The omission of such unobserved variables may introduce bias into the estimated effects of the included covariates. In particular, ocean currents have been shown to drive spatial variability in key environmental parameters such as sea temperature and salinity, thereby influencing the movement of fish populations toward more suitable habitats [41]. Future research will need to acquire and integrate high-resolution oceanographic data to enable a more comprehensive and ecologically refined understanding of hairtail distribution dynamics. 

## 5. Conclusions

The SDM was applied to investigate the spatial determinants of hairtail catch volumes in the southern coastal waters of South Korea, utilizing high-resolution quarterly grid-level data collected from 2020 to 2022. The analysis indicated that Sal exerted the strongest positive direct effect on catch volumes, while simultaneously exhibiting significant negative spillover effects on neighboring areas. DO consistently demonstrated negative effects both within and across regions, suggesting behavioral or physiological adaptations of hairtail to low-oxygen environments. Chl-a exhibited statistically significant positive effects in both direct and indirect dimensions, indicating that elevated primary productivity facilitates local aggregation and propagates beneficial effects to neighboring areas. SST also showed a statistically significant direct effect, but it was weaker than other variables. Additionally, fishing effort, measured by the number of vessels, exhibited a significant positive direct effect, emphasizing the role of human activities in shaping the spatial distribution of hairtail.

These findings demonstrate the value of spatial econometric approaches in fisheries research by capturing how marine environmental and anthropogenic factors influence fish catch through both localized and spillover effects. By employing a spatial framework that distinguishes between direct and indirect effects, the analysis offers a more nuanced understanding of the ecological processes underlying hairtail distribution than conventional non-spatial methods. This spatial perspective is especially valuable in ecologically dynamic regions, such as the Jeju Strait, where seasonal variability, oceanographic fronts, and current systems converge.

## Figures and Tables

**Table 1:** Summary statistics of data.

Variable	Quarter	Observation	Mean	S.D.	Min.	Max.
Dependent variable						
Catch (Production, kg)	1	13,113	744	4944	0	241,902
	2	13,113	574	4124	0	305,075
	3	13,113	1506	5097	0	264,717
	4	13,113	3030	91,802	0	10,400,000
Independent variable						
Chl-a (Chlorophyll-a, mg/m^3^)	1	13,113	1.38	1.15	0.11	39.06
	2	13,113	1.38	1.82	0.06	58.65
	3	13,113	1.32	2.19	0.19	67.05
	4	13,113	1.36	1.64	0.24	49.76
DO (Dissolved oxygen, mg/L)	1	13,113	6.52	1.33	4.89	12.48
	2	13,112	6.44	1.09	4.84	10.04
	3	13,113	5.50	1.18	4.06	11.35
	4	13,112	5.54	1.09	3.95	10.08
Sal (Salinity, PSU)	1	13,113	33.86	0.84	28.03	35.21
	2	13,112	33.24	0.73	23.93	34.59
	3	13,113	31.10	0.62	19.85	33.99
	4	13,112	33.25	0.79	24.79	34.66
SST (Sea surface temperature, °C)	1	13,113	13.23	3.49	0.15	18.99
	2	13,113	17.87	2.98	0.14	21.88
	3	13,113	25.01	3.14	1.03	27.76
	4	13,113	18.40	3.41	1.22	23.40
Vessels (Vessel, vessel)	1	13,113	411	1077	0	28,800
	2	13,113	465	1168	0	26,056
	3	13,113	552	1633	0	58,578
	4	13,113	598	1542	0	45,675

Note: Parentheses in the table indicate the units of the variables; S.D., standard deviation; Min., minimum; Max., maximum.

**Table 2:** VIF estimation results.

Variable	VIF	1/VIF
Chl-a	1.96	0.51
DO	2.76	0.36
Sal	1.31	0.76
SST	1.95	0.51
Vessels	1.65	0.61

**Table 3:** Hausman test results: fixed vs. random effects.

Variable	FE	RE
Chl-a	0.25	−0.92
DO	−10.17	−5.59
Sal	0.60	−1.09
SST	0.56	1.03
Vessels	0.57	0.69
Hausman Chi-squared (*p*-value)	1641.74 *** (0.00)	

Note: * *p* < 0.1, ** *p* < 0.05, *** *p* < 0.01; FE, fixed-effects model; RE, random-effects model.

**Table 4:** Spatial autocorrelation analysis results—hairtail.

Matrix	Quarter	Moran’s I	Chi-Squared	Z-Value
Inverse Distance	1	0.089	372.26	19.29 ***
	2	0.149	1199.24	34.63 ***
	3	0.444	8930.63	94.50 ***
	4	0.003	8.20	2.86 ***

Note: * *p* < 0.1, ** *p* < 0.05, *** *p* < 0.01.

**Table 5:** Akaike’s information criterion and Bayesian information criterion—SDM, SDEM model.

Model	AIC	BIC
SDM	178,278.60	178,385.00
SDEM	177,077.80	177,184.20

**Table 6:** Results of panel models.

Variable	Panel Model	SEM	SAR	SLX	SDM
	Coefficient (t-Statistic)	Coefficient (z-Statistic)	Coefficient (z-Statistic)	Coefficient (z-Statistic)	Coefficient (z-Statistic)
Chl-a	0.25 *** (3.14)	−0.37 *** (−4.42)	−0.72 *** (−9.77)	−0.35 *** (−3.46)	0.62 *** (6.91)
DO	−10.17 *** (−51.34)	−1.22 *** (−2.87)	5.17 *** (20.38)	4.56 *** (12.91)	−5.82 *** (−17.88)
Sal	0.60 (1.23)	−0.55 (−0.63)	1.66 *** (3.74)	−1.22 (−1.11)	14.43 *** (14.53)
SST	0.56 *** (7.17)	1.15 *** (10.67)	−0.22 *** (−2.99)	1.33 *** (10.90)	0.46 *** (4.23)
Vessels	0.57 *** (34.34)	0.58 *** (31.98)	0.47 *** (31.20)	0.53 *** (24.27)	0.53 *** (27.26)
W*Chl-a				−2.08 *** (−2.78)	−15.01 *** (−22.17)
W*DO				−27.86 *** (−40.25)	61.50 *** (65.96)
W*Sal				−2.61 (−1.31)	−39.93 *** (−22.09)
W*SST				−5.12 *** (−12.93)	−1.90 *** (−5.35)
W*Vessels				1.00 *** (8.72)	−2.32 *** (−21.99)
Constant	16.75 *** (8.98)				
ρ			1.54 *** (86.51)		4.18 *** (127.99)
λ		1.25 *** (0.02)			
log-likelihood	−94,900.00	−91,730.00	−91,500.00	−93,540.00	−89,130.00
*n*	52,452	52,452	52,452	52,452	52,452

Note: * *p* < 0.1, ** *p* < 0.05, *** *p* < 0.01; SAR, Spatial Autoregressive Model; SEM, Spatial Error Model; SDM, Spatial Durbin Model; In the variable, the asterisk (*) (e.g., W*Chl-a) denotes the spatial lag, representing the application of the spatial weight matrix (W) to the variable.

**Table 7:** Likelihood ratio and Wald test results for reducing the SDM to SLX, SAR, panel, and SEM models.

Test		$H_{0}$	$H_{1}$	$\chi^{2}$ (df)
	SDM vs. SLX	ρ=0	ρ≠0	8826.61 *** (1)
LR	SDM vs. SAR	θ=0	θ≠0	4744.32 *** (5)
	SDM vs. Panel	ρ=0,θ=0	ρ≠0 or θ≠0	11,541.26 *** (6)
Wald	SDM vs. SEM	θ+ρβ=0	θ+ρβ≠0	1792.58 *** (5)

Note: * *p* < 0.1, ** *p* < 0.05, *** *p* < 0.01.

**Table 8:** Results of marginal effects for SDM.

Variable	Direct	Indirect	Total
	Coefficient (z-Statistic)	Coefficient (z-Statistic)	Coefficient (z-Statistic)
Chl-a	0.60 *** (6.72)	3.93 ** (22.88)	4.53 *** (22.85)
DO	−5.76 *** (−17.78)	−11.76 *** (−35.28)	−17.53 *** (−115.70)
Sal	14.47 *** (14.49)	−6.44 *** (−6.87)	8.03 *** (21.55)
SST	0.46 *** (4.21)	−0.01 (−0.07)	0.45 *** (4.64)
Vessels	0.53 *** (27.22)	0.04 (1.44)	0.56 *** (19.71)

Note: * *p* < 0.1, ** *p* < 0.05, *** *p* < 0.01.

## Data Availability

The original data presented in this study are openly available in references [19,20,21]. Some of these data are not publicly available or stored elsewhere due to ethical and privacy issues. Some anonymous data collected in this study can be requested from the corresponding author, although its availability will require the participants’ consent.

## Abbreviations

The following abbreviations are used in this manuscript:

AIC	Akaike Information Criterion
BIC	Bayesian Information Criterion
Chl-a	Chlorophyll-a
DO	Dissolved Oxygen
FE	Fixed Effect
LR	Likelihood Ratio
OLS	Ordinary Least Squares
RDA	Redundancy Analysis
RE	Random Effect
Sal	Salinity
SAR	Spatial Autoregressive Model
SDEM	Spatial Durbin Error Model
SDM	Spatial Durbin Model
SEM	Spatial Error Model
SST	Sea Surface Temperature
VIF	Variance Inflation Factor
